# From Patient Selection to Surveillance: Artificial Intelligence Applications in Radioiodine Therapy – A Systematic Review

**DOI:** 10.1007/s13139-026-01049-w

**Published:** 2026-07-11

**Authors:** Maria Obrycka, Saeid Abdelrazek, Łukasz Żukowski, Janusz Myśliwiec, Piotr Szumowski

**Affiliations:** 1 Department of Nuclear Medicine, Medical University of Bialystok, Maria Skłodowska-Curie 24 A St., 15276 Bialystok, Poland; 2 Doctoral School, Medical University of Bialystok, Jana Kilińskiego 1 St., 15089 Bialystok, Poland

**Keywords:** Artificial intelligence, Radioiodine, ^131^I, Hyperthyroidism, Differentiated thyroid cancer

## Abstract

Radioactive iodine therapy requires individualized decisions regarding patient selection, administered activity planning, imaging assessment, and follow-up. This systematic review summarizes current applications of artificial intelligence (AI) in the ^131^I treatment pathway and evaluates barriers limiting clinical implementation. Following PRISMA 2020 guidelines, PubMed, Scopus, Web of Science, and Wiley databases were searched for original studies involving human patients treated with ^131^I for differentiated thyroid cancer or benign thyroid disease, where AI, deep learning (DL), or machine learning (ML) methods were utilized. Evidence was synthesized, and study quality was assessed using the NIH/NHLBI Study Quality Assessment Tool. Among 775 identified records, 24 studies met inclusion criteria. AI applications included pretreatment decision support, detection and characterization of iodine-avid disease, dosimetry and activity optimization, radiation-safety assessment, treatment response prediction, levothyroxine dose estimation, and recurrence-risk stratification. The available evidence was dominated by retrospective, single-center studies, primarily focused on differentiated thyroid cancer, with limited external validation. AI has the potential to improve standardization and personalization of ^131^I therapy, but heterogeneous methodologies and insufficient evidence of clinical utility currently restrict routine adoption. Prospective multicenter studies with harmonized endpoints and robust validation are required to facilitate clinical translation.

## Introduction

### Radioactive Iodine Therapy: Basic Principles

Radioactive iodine therapy (RAIT) represents one of the earliest and most successful examples of targeted radionuclide therapy in medicine, leveraging the unique ability of thyroid follicular cells to actively concentrate iodide via the sodium–iodide symporter (NIS) [1–5]. Since its first clinical application in the early 1940 s by Saul Hertz [6], RAIT has become a cornerstone in the management of both benign and malignant thyroid diseases [7–10]. Despite ongoing debate regarding the potential risk of secondary malignancies following RAIT, the available evidence remains heterogeneous and inconclusive [10–13]. While several large studies have not demonstrated a clear increase in overall cancer-related mortality or heritable genetic effects after therapeutic ^131^I exposure [14], recent meta-analyses have reported possible dose-dependent associations with specific secondary malignancies, including leukemia and salivary gland cancers [15–17]. Additionally, emerging evidence suggests that RAIT may be associated with clonal hematopoiesis, a potential precursor of hematologic malignancies, although the long-term clinical significance of this finding remains uncertain [18]. Despite RAIT being generally safe and well tolerated, with most adverse effects being mild and short-term (including radiation thyroiditis, post-therapy thyrotoxicosis, salivary gland dysfunction, transient bone marrow suppression, hypothyroidism, and Graves’ orbitopathy) [19, 20], current long-term evidence remains inconclusive, highlighting the need for continued long-term surveillance and individualized risk–benefit assessment, particularly given the high disease burden [9, 21–23].

### RAIT Challenges

#### Activity Selection

One of the central challenges in RAIT is selecting the administered activity, as it directly determines the balance between therapeutic efficacy and radiation-related toxicity. However, no universal consensus exists regarding the optimal administered activity [24–28]. Instead, major societies typically recommend activity ranges tailored to clinical intent (e.g., remnant ablation, adjuvant therapy, or treatment of known metastatic disease), which contributes to meaningful variation in real-world practice [10, 29]. In differentiated thyroid cancer, activity selection is influenced by the method of TSH stimulation (thyroid hormone withdrawal vs rhTSH) [1]. Pre-therapy iodine status also significantly affects ^131^I biodistribution, and low-iodine diets are routinely used to maximize uptake in target thyroid tissue [30]. Whole-body radiation exposure depends largely on systemic iodine retention and clearance, which may be prolonged in conditions of renal impairment [31]. Similarly, lithium can increase absorbed dose to target tissue by prolonging ^131^I retention, but may also increase non-target radiation exposure, requiring careful dosimetric assessment [1, 32].

In benign disorders, the Marinelli formula is used to calculate approximate target absorbed dose, although it requires accurate volume and uptake data, and doesn’t explicitly account for clinical history such as antithyroid drug (ATD) use, which can change iodine kinetics [33]. Furthermore, the short-interval intraindividual variability in Graves’ uptake/turnover and phenomena such as stunning or self-stunning complicate dose prediction, such that “meticulous dosimetry” still may not achieve a precise individual thyroid dose [34]. Clinically, centers therefore use either standardized (fixed) activities, sometimes roughly adapted to thyroid size, or dosimetric calculations based on thyroid volume and biokinetics often using the Marinelli approach to approximate a target absorbed dose.

#### Radioactive Iodine and Imaging

Planar thyroid radionuclide imaging is limited by subjective interpretation, reader-dependent variability, tissue superposition, and limited quantification [35]. SPECT/CT improves lesion localization and quantitative assessment of ^131^I uptake, but requires hybrid equipment, increases cost and complexity, and adds CT-related radiation exposure [36]. Moreover, the lack of widely implemented automated thyroid segmentation and the continued reliance on manual image processing prolong analysis time and introduce substantial inter-operator and inter-center variability [37, 38]. Thyroid ultrasonography is widely accessible, safe, and relatively inexpensive with high spatial resolution, but it does not provide direct functional information on iodine uptake or organ kinetics [39]. Notably, radiology has increasingly addressed analogous challenges such as reader variability, time-intensive segmentation, and standardization of quantitative reporting through AI-enabled tools for automated detection, segmentation, and workflow triage, which have demonstrated improved efficiency and reproducibility in several imaging domains [40].

These advances suggest that nuclear medicine could similarly benefit from AI-driven solutions, particularly for automated segmentation, quantitative analysis, and CT-free attenuation correction in thyroid SPECT, thereby improving standardization, reproducibility, and clinical scalability of imaging in the context of RAIT.

#### Predicting RAIT Outcomes

Individual-level outcome prediction after RAIT is inherently complex in both benign thyroid disease and post-thyroidectomy differentiated thyroid cancer, because therapeutic response emerges from multiple interacting determinants that are rarely measured comprehensively before administration. Reported predictors of RAIT failure in benign hyperthyroidism include male sex, delayed RAIT, prior ATD use, higher fT4, very high 24-h uptake, and larger thyroid volume [41], but key modifiers (ATD timing/duration, iodine contamination after contrast) are often inconsistently measured or documented [42, 43]. In DTC, prognostication is further limited by heterogeneity in remnant or tumor burden and especially lesion-level iodine avidity, and higher prescribed activities do not reliably improve long-term outcomes [44]. In benign hyperthyroidism, clinically relevant outcomes such as remission, persistent hyperthyroidism, or post-therapy hypothyroidism may differ despite similar activity-selection strategies [45]. Reported success rates therefore vary considerably between studies due to differences in outcome definitions and follow-up duration, as thyroid function may continue to change for months after ^131^I therapy [46–48]. These findings highlight the need for standardized endpoints and follow-up periods when evaluating post-RAIT outcomes and support the development of integrated predictive models combining clinical exposure history, imaging, and biokinetics.

### Artificial Intelligence in Nuclear Medicine

**Fig. 1:**
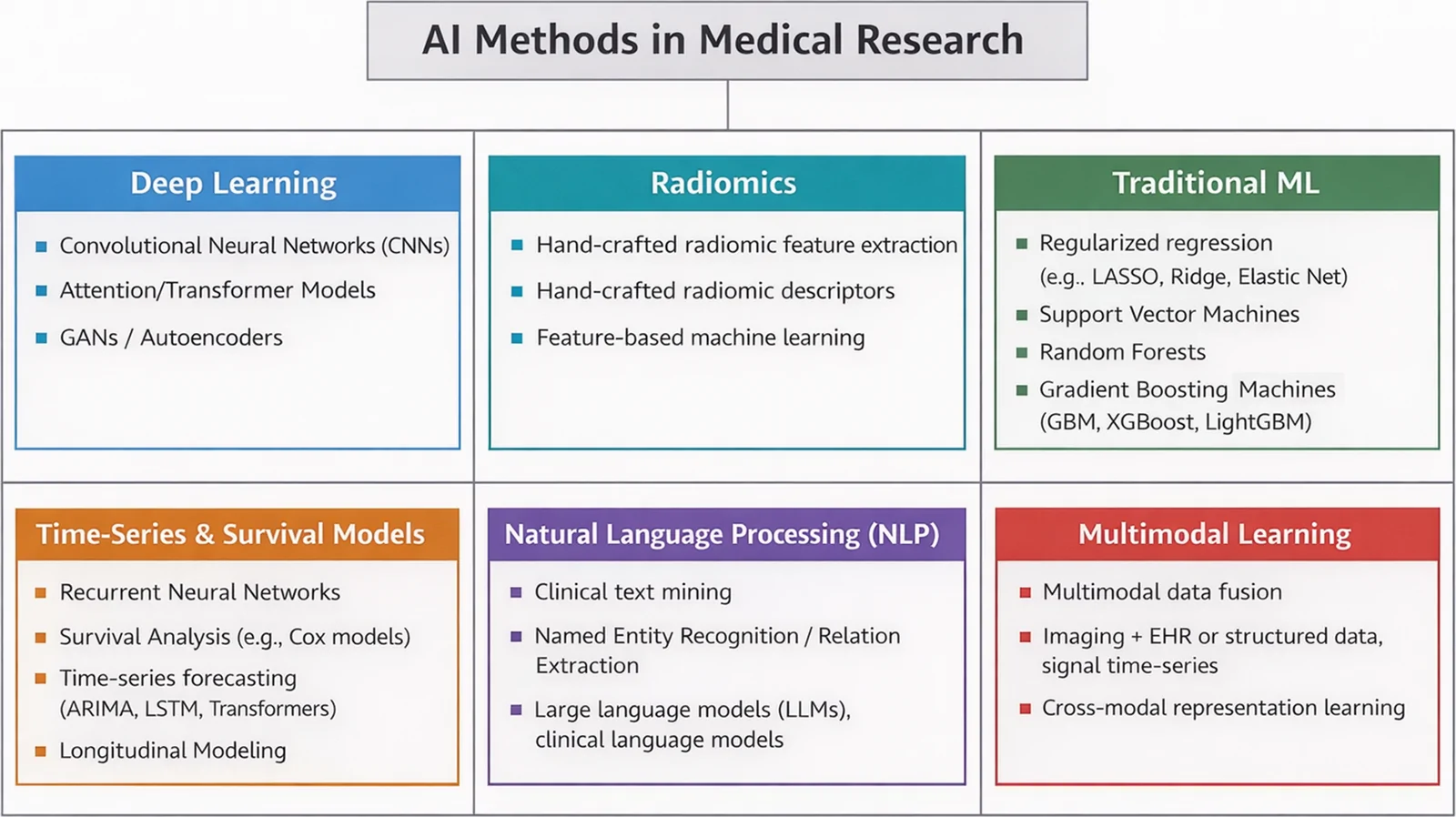
AI methods in medical research

Methodologically, clinical AI applications can be categorized according to data modality and intended clinical function (Fig. 1). In medical imaging, DL models are widely used for classification, detection, and segmentation tasks, enabling automated quantitative analysis and imaging biomarker extraction [49, 50]. Complementary radiomics approaches use hand-crafted imaging features combined with classical ML algorithms to improve interpretability and integration with clinical data [51]. Structured clinical and laboratory data are commonly analyzed using supervised learning models for risk prediction and treatment-response assessment, while natural language processing enables extraction of information from unstructured clinical records.

The complexity and variability of radioactive iodine (^131^I) therapy make it a promising area for AI-supported clinical decision-making [49, 50]. AI methods may help address some obstacles by integrating multimodal data to support more precise and individualized therapeutic decisions [51, 52]. DL approaches could improve automated image analysis and quantitative assessment, while ML models may enhance prediction of treatment response, absorbed dose estimation, and risk stratification [52]. Importantly, AI may assist in addressing the shortage of nuclear medicine specialists by serving as a decision-support and educational tool for trainees, improving interpretation skills and clinical consistency [53–56]. Clinical implementation, however, requires high-quality, curated data, rigorous validation, calibration, interpretability, and clinically relevant performance metrics to ensure safety and generalizability across populations [56–60].

## Materials and Methods

### Contents of Review

This systematic review advances the field of artificial intelligence applications in RAIT by critically synthesizing evidence on AI-supported diagnostic assessment, dosimetry, treatment response prediction, and safety/logistics considerations, while addressing key methodological, clinical, and ethical challenges. Unlike previous reviews focused mainly on model performance, this work adopts a clinically oriented perspective linking AI developments with real-world nuclear medicine workflows and decision-making. The review provides a taxonomy of AI applications in RAIT, identifies major evidence gaps such as heterogeneous datasets and limited external validation, highlights AI as a decision-support tool, discusses ethical and governance considerations, and outlines priorities for future research, including an explainable AI and prospective clinically integrated validation studies.

### Formalization of Research Questions

This systematic review aims to classify current AI approaches applied to RAIT, critically appraise their reported performance and clinical utility, identify evidence gaps, and outline priorities for future research to support safe, scalable, and ethically responsible translation into clinical nuclear medicine practice. To guide the review, we formulated the following research questions:

- **RQ1:** How can AI methods used in the context of RAIT be systematically categorized according to clinical tasks (e.g., imaging interpretation/quantification, response prediction, dosimetry/activity planning, radiation safety and logistics) and modeling approach?
- **RQ2:** What data sources (e.g., SPECT/planar imaging, whole-body scintigraphy, post-therapy scans, clinical and laboratory variables, dosimetry/biokinetic measurements) and validation strategies (e.g., internal vs external validation, single-center vs multicenter, retrospective vs prospective designs) are most commonly used?
- **RQ3:** What methodological and clinical trends emerge from the included studies (e.g., dominant AI paradigms, most frequently targeted endpoints such as ablation success or treatment response, and the distribution of applications across thyroid cancer vs benign thyroid disease)?
- **RQ4:** What are the principal barriers to clinical implementation of AI in RAIT, and what future directions are most likely to improve generalizability, interpretability, safety, and governance (including bias mitigation and human–AI decision support)?

The prespecified research questions are addressed throughout the *Results* by extracting study-level evidence relevant to each question, and are subsequently synthesized in the *Discussion* section.

#### The Process of Selecting Papers

To provide an overview of AI methods used during the RAIT process, we conducted a systematic review using four databases: PubMed, Scopus, Web of Science and Wiley. The search was conducted using database-specific syntax that combined controlled vocabulary terms with keywords related to AI applications in RAIT. The search formula was:

$$\begin{array}{c}\big(\big(\mathrm{prognosis}\big)\mathrm{OR} \big(\mathrm{(prediction}\big) \mathrm{OR}\big(\text{(side effects}\big)\mathrm{OR} \big(\mathrm{outcome}\big)\\\mathrm{OR} \big(\mathrm{analysis}\big)\big) \mathrm{AND} \big(\big({}_{\hspace{0.17em}}{}^{131}\mathrm{I}\big) \mathrm{OR} \big(131-\mathrm{I}\big) \mathrm{OR} \big(\mathrm{I}131\big)\\\mathrm{OR} \big(\mathrm{radioiodine}\big)\big)\mathrm{AND} ((\text{artificial intelligence}) \mathrm{OR} (\text{machine learning}) \\ \mathrm{OR} (\text{deep learning}) \mathrm{OR} (\mathrm{CNN}) \mathrm{OR} (\text{neural network}))\end{array}$$

**Fig. 2:**
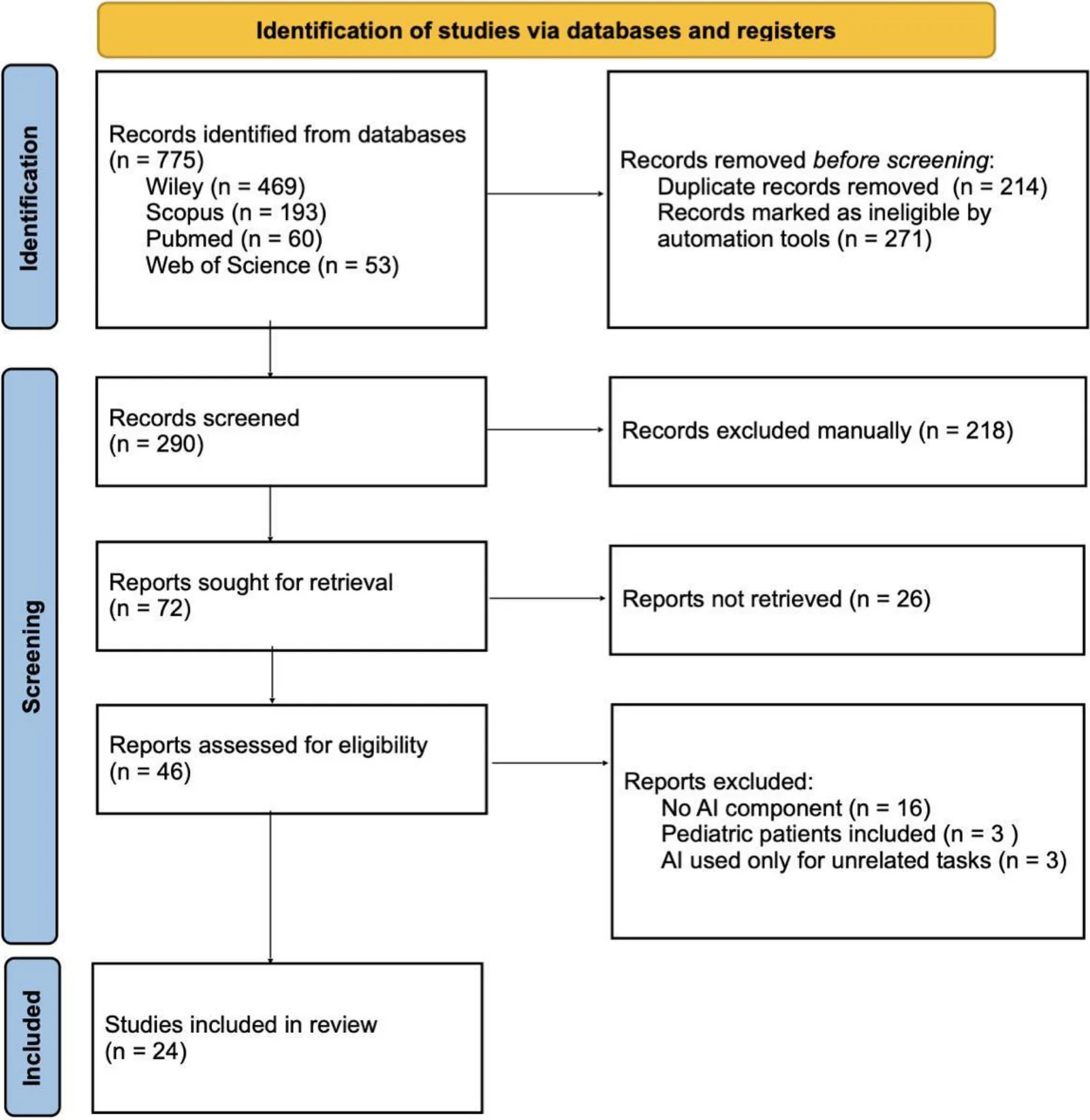
PRISMA 2020 flow diagram [61]

Efforts were made to prevent duplicate or overlapping data, with duplicate records initially removed automatically using Zotero software (Version 7.0.20; Corporation for Digital Scholarship, Vienna, Virginia, USA) and subsequently verified through manual review. Titles and abstracts were screened manually with full-text articles reviewed to confirm eligibility based on inclusion and exclusion criteria established using the PICO (Population, Intervention, Comparison, Outcome) framework, as presented in Table 1. Detailed search protocol according to the guidelines of the Preferred Reporting Items for Systematic Reviews and Meta-Analyses 2020 (PRISMA 2020) framework [61, 62] is presented in section “Results” in Fig. 2. The protocol for this systematic review was registered in the International Prospective Register of Systematic Reviews (PROSPERO) under registration number CRD420261397734.

**Table 1:** Inclusion and exclusion criteria shown using the PICO framework

Category	Inclusion criteria	Exclusion criteria
Population (P)	Human adult subjects with thyroid disease treated with ^131^I: DTC (remnant ablation/adjuvant therapy/metastatic disease) or benign disease (Graves’ disease, toxic adenoma, toxic multinodular goiter, non-toxic goiter)	Non-human studies, pediatric population studies, purely simulation-based studies without clinical patient data
Intervention/Index (I)	Use of AI/ML/DL methods applied to RAIT workflows: imaging interpretation, segmentation, outcome prediction, dosimetry, safety, discharge decisions	Studies not involving AI methods or AI used only for tasks unrelated to RAIT
Comparator (C)	Conventional statistical methods, standard clinical assessment, clinician- or expert-based interpretation, established clinical scoring systems; where no external comparator was available, studies were included if internal validation procedures and model performance metrics allowed assessment of model performance and potential clinical relevance	No comparator or evaluable reference provided
Outcomes (O)	Reports at least one clinically relevant endpoint: treatment response, ^131^I-refractory status, adverse effects, dosimetry, exposure to contacts, discharge time	No measurable outcomes
Study type	Original research with patient-level or imaging-level clinical data (prospective or retrospective), including technical studies validated on clinical datasets	Reviews, editorials, letters, protocols without results, conference abstracts without full text, case reports
Topic scope	Thyroid-related (benign or malignant) with AI contribution to clinical decision-making or quantification	Nuclear explosion outcome prediction, environmental, weapon-related fallout modeling, isotope decay theory, papers unrelated to RAIT
Language/access	English full text	Non-English without accessible translation or inaccessible full text

#### Inclusion and Exclusion Criteria

We included studies involving adult patients treated with ^131^I for differentiated thyroid cancer or benign thyroid disorders (e.g., Graves’ disease, toxic adenoma, toxic multinodular goiter) that applied AI methods such as ML, DL, or CNN-based approaches to clinically relevant components of RAIT, including imaging, outcome prediction, dosimetry, radiation safety, or discharge planning. Non-human, theoretical, unrelated isotope studies, reviews, editorials, conference abstracts, protocols without results, and studies lacking measurable RAIT-related outcomes were excluded.

#### Data Extraction and Synthesizing the Studies

We applied a prespecified, standardized extraction template to capture study-level evidence consistently, including bibliographic context, clinical use case (imaging/segmentation, response prediction, dosimetry/activity planning, radiation safety/logistics), study design and cohorts, data modalities, and the full AI pipeline. We also extracted validation approaches, performance metrics, and author-reported limitations relevant to bias and generalizability. Two reviewers performed independent extraction with discrepancies resolved by consensus meetings. Synthesis was organized by application domain and used narrative and thematic analysis to identify methodological patterns, sources of heterogeneity, and translational barriers; given expected heterogeneity in populations, endpoints, and reporting, meta-analysis was not prioritized, with emphasis placed on rigor, clinical relevance, and reproducibility.

## Results

The literature review identified 775 records in databases. Zotero automatically identified 214 duplicate records and 271 records were excluded based on their publication type (e.g. editorials, conference abstracts, letters), which were subsequently verified by reviewers and removed. A total of 290 papers were eligible for screening. 218 records were excluded manually by two reviewers in agreement based on titles and abstracts, 26 reports were not retrieved in full-text availability. Out of 46 remaining papers, 24 were chosen based on previously established PICO criteria and were included in this review paper (see Fig. 2). The studies included in the review were published between 2018 and 2025. Discrepancies regarding the selection of studies for inclusion in the review were resolved through consensus meetings, while unresolved cases were evaluated and excluded by a senior nuclear medicine specialist. The detailed characteristics of included studies are provided in Table 2.

**Table 2:** Characteristics of included studies [63–86]

Author, Year	Study type	Setting	Diagnosis	Study aim	Primary cohort	Validation cohort	Method of AI used	Key performance metrics
Su et al., 2025	Retrospective single center	China	DTC treated with ^131^I	Predict occult lung metastases	371 DTC patients; occult lung meta (n = 55), T1 (n = 13), T2 (n = 17), T3 (n = 5), T4 (n = 20), N0 (n = 5), N1 (n = 50); 316 no meta; T1 (n = 218), T2 (n = 56), T3 (n = 5), T4 (n = 37), N1 (n = 209)	Internal validation by test:train 7:3 split (260:111)	Tabular ML (SVM, DT, RF, LR, XGBoost, KNN) on 11 clinical and lab variables; explainability with SHAP	Best model LR: AUC 0.93, accuracy 0.91, specificity 0.96
Gao et al., 2024	Retrospective multicenter	China	DTC with lung metastases with chest CT and SPECT post-RAIT	Predict non–iodine-avid lung metastases to support RAIT selection	496 patients, total of 1962 lung meta; ^131^I-avid (n = 318): nodule max diameter = 8.50 ± 5.68 mm; ^131^I-refractory (n = 178): nodule max diameter = 8.87 ± 4.35	External validation on 24 patients, 123 lesions; ^131^I-avid (n = 11): nodule max diameter = 6.4 ± 2.45 mm; ^131^I-refractory (n = 13): nodule max diameter = 6.83 ± 2.52 mm	Two-stage deep learning: automated lesion segmentation with SE V-Net, followed by lesion-level SE-Net classification to predict non–iodine-avid metastases	AUC 0.879 internal; 0.713 external; small nodules (≤ 4 mm) AUC 0.783; decision-curve analysis suggested net benefit
Song et al., 2025	Retrospective multicenter, population-based	China	DTC with lung metastases	Predict ^131^I uptake phenotype (avid vs non-avid) of lung metastases before therapy	261 patients; Avid groups (n = 140): PTC (n = 108), FTC (n = 32), lung-only meta (n = 128), lung + other meta (n = 12); Non-avid (n = 121): PTC (n = 95), FTC (n = 26), lung-only metas (n = 107), lung + other meta (n = 14)	Avid groups: Internal validation (n = 36), External validation 1 (n = 25), External validation 2 (n = 23); Non-avid groups: Internal validation (n = 23), External validation 1 (n = 18), External validation 2 (n = 18)	Two-stage CT pipeline: nodule detection (Faster R-CNN + FPN with parallel multitask RoI head) and later classification with ResNeSt50 (vs InceptionV3, ResNet50)	ResNeSt50 AUC: 0.722 internal, 0.720 external-1, 0.731 external-2
Georgiou et al., 2024	Retrospective single-center	United States of America	Adult DTC undergoing ^131^I dosimetry	Predict maximum permissible activity using only early time points (4/24/48 h) vs full protocol (4/24/48/72/96 h)	Cohort of 83 patients, Average Calculated Maximum Permissible Activity = 16872 MBq (range 4329–39 960 MBq)	Internal validation on random patients (n = 13) from primary cohort	Feedforward deep-learning NN (Keras); 8 inputs incl. normalized A/P counts + blood counts (4/24/48 h), age, weight; 2 hidden layers	No significant difference vs MIRD MPA on test set (*p* = 0.351); differences generally within ± 10%
Jalilifar et al., 2025	Retrospective single-center	Iran	Thyroid cancer (after thyroidectomy and ^131^I -NaI) + NET (^131^I-MIBG)	Approximate Monte Carlo organ absorbed doses from accessible planar imaging	Cohort of 70: thyroid cancer (n = 40) and NET (n = 30); no detailed characteristics	External validation on cohort of 23 patients; no detailed characteristics	ML/DL comparison; best model MLP (others tested incl. linear regression, SVR, decision tree; CNN/U-Net evaluated then excluded)	MLP achieved < 15% difference vs MC for most tissues, external validation showed no significant differences for liver/kidneys/thyroid, but there are larger negative bias for spleen (14.84%) and lung regions (13%)
Ciucci et al., 2024	Methodological feasibility study	Italy	mDTC treated with [^131^I]I-NaI	Improve TAC model selection (MEf vs BEf) and estimation of time-integrated activity (τ/AUC) under sparse/noisy sampling	Synthetic: train (n = 10,000) and test (n = 2000) with 5% noise; no detailed characteristics	External validation on cohort of 20 patients’ TACs	ML1: soft-voting ensemble of 2 logistic regressions (MEf vs BEf); ML2: AdaBoost ensemble of 5 gradient-boosting regressors (each built from 1000 decision-tree regressors)	Model classification accuracy ~ 98%; τ (AUC) error constrained to ± 25%; analytical pipeline underestimation down to ~ 66.7%; patient TACs: agreement R^2^ = 0.93, median difference ~ 0.2%
Popovic et al., 2021	Retrospective single-center + educational evaluation	Serbia	Papillary thyroid carcinoma after thyroidectomy without distant mets	Develop and evaluate the performance of three ML algorithms that suggest whether these patients should undergo RAIT and propose an administrable ^131^I dose	220 patients: T1a (n = 108), T1b (n = 52), T2 (n = 29), T3a (n = 5), T3b (n = 11), T4a (n = 5); multifocality (n = 101); extrathyroidal extension: none (n = 153), minimal (n = 42), gross (n = 15); N0 (n = 85), N1a (n = 46), N1b (n = 9), N1a + N1b (n = 71)	Internal validation by train:test split of cohort 150:70	Compared ANN vs Naïve Bayes vs GMDH	ANN was reported as the best: test accuracy 95.71%; weighted κ = 0.96 vs expert; junior-physician agreement improved κ from 0.70 to 0.93 after ANN-assisted learning
Ebrahimi et al., 2018	Retrospective single-center on dosimetry dataset	Iran	Thyroid cancer treated with 3700–7400 MBq	Develop and validate a feed-forward neural network to estimate dose to relatives following RAIT	Thyroid cancer patients (n = 52) and patient–relative pairs (n = 99); no detailed characteristics	Not reported	Neural Networks in this study were feed-forward with sigmoid activation function and one hidden layer with 13 inputs (administered/residual activity, discharge dose rate at 1 m, biokinetics/clearance indicators, occupancy/household context, anthropometrics, demographics)	Relative doses generally low (mean 0.45 mSv; median 0.28 mSv; range 0.1–3.64 mSv); children < 10 years higher; 7/99 > 1 mSv, none > 5 mSv; NN improved prediction vs conventional calculation (MSE 0.142 vs 1.060) and better identified > 1 mSv (ROC AUC 0.957 vs 0.633)
Zhang et al., 2025	Retrospective single-center	China	Thyroid cancer patients receiving RAIT	Predict 48 h discharge eligibility under radiation-safety threshold	1044 thyroid cancer patients: 5550 MBq (n = 852), 3700 MBq (n = 153), 7400 MBq (n = 39)	Not reported	Support Vector Machine (regularization parameter = 1) using routine variables (sex, age, administered activity, metabolism/residual-activity features); exponential fit used to estimate effective half-life	Performance: AUC 0.92, accuracy 88.04%, precision 96.89%, sensitivity 88.64%, F1 0.93
Lin et al. 2021	Retrospective multicenter	China	Postoperative thyroid carcinoma, bone metastasis, arthritis	Develop tool to automatically classify whole-body scintigraphic images of concern (i.e., the normal, metastasis, arthritis, and thyroid carcinoma)	1078 whole-body scintigraphy images from 600 patients: normal (n = 170), bone metastasis (n = 117), arthritis (n = 143), thyroid carcinoma (n = 161)	Internal validation by train:test split of cohort 7:3	Attention-augmented CNN, Hybrid attention module: channel attention + spatial attention, supervised learning with physician-labeled ground truth	Attention-augmented CNN (Dscint) achieved accuracy/precision/recall/F1 = 0.9801/0.9795/0.9791/0.9792 on the augmented test set (AUC 0.9985), compared with accuracy/F1 = 0.8519/0.8362 on the original dataset; among baselines, Dscint outperformed AlexNet (accuracy/F1 0.9652/0.9715) and VGG-16 (accuracy/F1 0.9550/0.9541), while Inception-v4 performed worst (accuracy/F1 0.8384/0.8345)
Chantadisai et al. 2024	Retrospective single-center	Thailand	Low-risk PTC post thyroidectomy	Explore the feasibility of combined radiomics of post-therapy ^131^I total body scan and clinical parameter to predict successful ablation in low-risk PTC	130 patients, all received 1110 MBq ^131^I; successful ablation (n = 77), unsuccessful (n = 53); mean pre-ablative serum Tg = 10.54 (± 13.36) ng/ml; median follow-up time = 252 days; single segment of ROI (n = 113), two ROIs (n = 16), three ROIs (n = 1)	Internal validation on primary cohort, no detailed characteristics	Automated ROI segmentation (3D Slicer; max-entropy threshold + smoothing), PyRadiomics feature extraction (464 features), regularized logistic regression (Lasso/Ridge) + RFE, cross validation (repeat run 20 times)	AUC (train/val): Tg-only 0.66/0.65, radiomics 0.77/0.69, combined 0.87/0.78; Validation (acc/sens/spec): Tg-only 0.65/0.85/0.32, radiomics 0.66/0.79/0.46, combined 0.74/0.81/0.62
Lubin et al. 2021	Retrospective single-center	United States of America	DTC after thyroidectomy receiving first RAIT	Predict initial RAIT failure and identify predictors	107 patients: classic follicular cell PTC (n = 4), tall-cell variant (n = 1), Hürthle-cell (n = 1), Follicular cell PTC NOS (n = 99); time range from RAIT to follow-up = 9–40 months, mean = 15.2 ± 5.5 months; ^131^I administered activity range: 1102.6–7622 MBq, mean = 3881.3 ± 1975.8 MBq; Medical history: Hashimoto’s thyroiditis (n = 9), toxic multinodular goiter (n = 1), pheochromocytoma (n = 1), none/other (n = 96); Tumor spread at surgery: extension beyond thyroid (n = 41), internal to gland (n = 61), cervical lymph node positive (n = 10); lymph node surgical dissection (n = 44); failure (n = 46): by serum biomarkers (n = 35), by follow-up imaging (n = 11)	Internal validation on primary cohort, no detailed characteristics	Python scikit-learn (Python v2.7) to analyze 16 clinical variables, a random forest classifier (for categorical/non-continuous data), cross-validation and data shuffling to reduce the risk of overfitting, comparison against multivariate logistic regression in SPSS	Significant predictors (*p*; RR): Pre-RAIT Tg *p* < 0.0001, RR 2.26; Pre-RAIT TgAb *p* = 0.011, RR 1.82; Multifocality *p* = 0.026, RR 1.73; Extent at surgery (*p* = 0.0135, RR 1.914; Administered ^131^I dose > 5920 MBq: *p* = 0.0147, RR 2.12; Borderline: time from surgery to RAIT = 0.064 RR 0.78; tumor size ≥ 4.5 cm: *p* = 0.057
Sa et al., 2024	Retrospective single-center	China	DTC without structural disease undergoing RAIT and TSH suppression	Predict effective response to RAIT and biochemical remission under TSH suppression	Train cohort 597: PTC (n = 586), FTC (n = 11); T: T1 (n = 397), T2 (n = 98), T3 (n = 66), T4 (n = 36); N0 (n = 48), N1a (n = 215), N1b (n = 334); stage: I (n = 471), II (n = 122), III (n = 4), IV (n = 0); risk: low (n = 60), intermediate (n = 483), high (n = 54)	Test cohort n = 257: PCT (n = 253), FTC (n = 4); T1 (n = 167), T2 (n = 52), T3 (n = 24), T4 (n = 14); N0 (n = 14), N1a (n = 108), N1b (n = 135); stage I (n = 208), II (n = 48), III (n = 1), IV (n = 0); risk: low (n = 25), intermediate (n = 206), risk (n = 26)	Logistic Regression, Support Vector Machine, Random Forest (RF), Neural Networks, Adaptive Boosting, and Gradient Boost	RAIT “effective response” model: AUC 0.896, accuracy 81.3%; TSH-suppression “biochemical remission” model: AUC 0.857, accuracy 78.7%
Bülbül & Nak, 2024	Retrospective single-center	Turkey	Papillary thyroid carcinoma treated with ^131^I	Predict excellent response after RAIT	Cohort of ER (n = 118): largest tumor diameter (mm) = 19.8 ± 12.4, preablation sTg (ng/mL) = 5.3 ± 10.2, ^131^I dose 3589 ± 1184 MBq; poor-prognosis subtype (n = 37), multifocality (n = 86), positive surgical margin (n = 24), lymphatic invasion (n = 28), vascular invasion (n = 11), perineural invasion (n = 8), extrathyroidal extension (n = 4); ATA 2015 risk group: low (n = 61), intermediate (n = 53), high (n = 4); T1–T2 (n = 112); T3–T4 (n = 6); N0 (n = 102); N1a/N1b (n = 16); TgAb: positive (n = 34), negative (n = 82); time to RAIT: < 3 months (n = 87), ≥ 3 months (n = 31); remnant tissue on postablation WBS (n = 109)	Internal validation by train:test split 80:20	Built models in Orange Data Mining toolbox v3.34.0, class imbalance with SMOTE, tenfold cross-validation on the training set; features normalized to 0–1, selected the 10 best predictors by ANOVA, then trained multiple ML models: kNN, decision tree, random forest, gradient boosting, extreme gradient boosting (XGBoost), naïve Bayes, logistic regression, neural network, AdaBoost	On standard statistical comparison, the only clearly significant difference between ER and non-ER groups was pre-RAIT TgAb positivity; Other variables did not reach significance in their univariable comparisons. Pre-treatment TgAb positivity was observed in 29% of the ER group versus 55% of the non-ER group (*p* = 0.007). Eight machine-learning models demonstrated good discrimination for ER, each with AUC > 0.70. The top-performing model by AUC was extreme gradient boosting (AUC = 0.871), while gradient boosting delivered the highest accuracy (81%)
Liu et al., 2024	Retrospective model development with prospective two-center validation	China	Intermediate or high-risk differentiated thyroid cancer after total thyroidectomy + RAIT	Predict initial post-RAIT levothyroxine dose to reach ATA risk–adapted TSH targets faster	266 patients, Hashimoto’s thyroiditis (n = 71), cardiovascular or cerebrovascular disease: (n = 27), digestive system disease (n = 19), other malignancies (n = 4), family history of malignancy (n = 22); mean age: 41.24 ± 11.59, mean weight: 68.44 ± 12.92 kg, height: 166.47 ± 7.85 cm, BMI: 24.57 ± 3.45, BSA: 2.36 ± 0.19 m^2^, mean Hb: 139.51 ± 20.51 g/L, mean MCV: 86.92 ± 13.20 fL, mean blood pressure: 125.60 ± 18.86/82.68 ± 12.29 mmHg (systolic/diastolic), post-operative mean PTH: 3.67 ± 2.43 pmol/L	263 patients; Center 1 (n = 88); Center 2 (n = 175)	Random-forest–based feature selection (via variable importance and out-of-bag error), then eight regression algorithms (including linear/ridge/lasso, random forest regression, kNN regression, Poisson/gamma regression, and support vector regression), selecting the best model via fivefold cross-validation with a predefined dose-error criterion	SVR accuracy 53.4% (± 12.5 µg), 80.45% (± 25 µg); improved early TSH target rates (~ 1 mo 52.09% vs 10.53%; ~ 2 mo 85.55% vs 53.38%) and shorter time-to-target (62.61 vs 115.50 days) vs historical empirical dosing
Ngan et al., 2025	Retrospective single-center	Vietnam	Hypothyroidism (post-RAIT, post-surgical, autoimmune, drug-induced)	Estimate individualized daily levothyroxine dose using ML beyond simple weight-based dosing	1864 patients; Causes of hypothyroidism: post-surgical (n = 1094), post-RAIT hypothyroidism (n = 264), autoimmune (n = 150), drug-induced (n = 80), unknown (n = 276); median weight = 54.0 kg, height = 155.0 cm, BMI = 22.21, median blood pressure = 121/72 mmHg (SBP 121.0, DBP 72.0), concomitant diseases (n = 3); median daily levothyroxine dose was 62.5 µg; concomitant medications: multivitamins (n = 251), calcium supplements (n = 189), PPIs (n = 134), magnesium salts (n = 74), iron preparations (n = 64), potassium chloride (n = 59), alendronate (n = 31), ciprofloxacin (n = 4), and zinc (n = 6); median labs: AST 23.28 U/L (18.02–30.05; 10.01–157.13), ALT 24.66 U/L (21.22–31.05; 7.45–306.07), TSH 2.52 mIU/L (1.50–3.77; 0.05–6.81), serum albumin 44.10 mg/dL (33.23–54.13; 20.01–64.96)	Internal validation by train:test split of 80:20 (1491:373)	Multi-model regression comparison with hyperparameter tuning; best model Extra Trees Regressor; performance via MAE/RMSE/R^2^	Best model: R^2^ 0.8737; MAE 9.4 µg/day (95% CI 7.7–11.2)
Duan et al. 2022	Retrospective single-center	China	Graves’ disease treated with ^131^I	Predict early hypothyroidism at 6 months after ^131^I to support pre-therapy risk stratification and post-treatment monitoring	471 patients; median duration of hyperthyroidism = 12 months; thyroid texture: hard (n = 172), soft (n = 299); thyroid nodule presence (n = 126); ATDs (n = 357), median ATDs duration (months) = 5; presence of eye disease (n = 105), hyperthyroid cardiomyopathy (n = 64), abnormal liver function (n = 141), leucopenia (n = 157), diabetes (n = 52), hypokalemic periodic paralysis (n = 23)	Internal validation by train:test split of cohort (310:160)	ML feature selection using random forest importance (1000 trees), PLS-DA VIP scores, and Mann–Whitney testing; final 7-feature multivariable model (age, thyroid mass, 24-h RAIU, AST, TRAb, TMA, neutrophils) presented as a nomogram	AUROC 0.72 (training) and 0.74 (validation); accuracy approximately 71–74%; 69.4% developed hypothyroidism at 6 months
Wang et al. 2025	Retrospective database, single-center	China	Hyperthyroidism	Automate thyroid CT segmentation with uncertainty quantification and enable patient-specific absorbed-dose assessment for ^131^I using MIRD plus Monte Carlo, compared with Marinelli–Quimby	HaN-Seg database, CTs of thyroid, brainstem, glottis, supraglottic region, and spinal cord, n = 5888 images	Test cohort of (n = 10) patients with hyperthyroidism	DL CT thyroid segmentation with uncertainty quantification (MSRA-UNet + +), integrated into MIRD absorbed-dose assessment with Monte Carlo (MCNP) S values	HaN-Seg thyroid segmentation: DSC 88.23%, HD95 9.43 pixels; clinical cohort: DSC 85.61% and Jaccard 75.16% (vs UNet + + baseline); Marinelli–Quimby dose overestimation 7.74% on average (max 10.73%) vs MIRD/Monte Carlo
Wang et al., 2024	Retrospective single-center	China	PTC after thyroidectomy + RAIT	Predict structural recurrence and compare with ATA risk stratification	2244 patients, median follow-up = 45.5 months, patients experienced structural recurrence (n = 179); median tumor diameters of ≤ 10 mm (n = 1093), 10 to 20 mm (n = 777), 20 to 40 mm (n = 322), > 40 mm (n = 52); multifocality (n = 577); extensive ETE (n = 584); median non-stimulated Tg = 0.24 ng/mL; median TSH = 0.46µIU/ml; central LN dissection (n = 1537) lateral LN dissection (n = 698); N1a (n = 1173), N1b (n = 564); median LNR = 28.57%; low risk (n = 328), intermediate risk (n = 1334), high risk (n = 582)	Internal validation by train:test split of 4:1 (1795:449)	Tabular ML (LR/SVM/XGBoost/RF/NN) + Spearman filtering + LASSO; imbalance handled with OSS under-resampling; fivefold CV + Bayesian HPO	Test AUC range 0.738–0.767 vs ATA 0.620; best discrimination NN AUC 0.767, RF 0.766; RF accuracy 0.775
Kim et al., 2021	Retrospective single-center	Korea	DTC, post–total thyroidectomy + RAIT; > 5y follow-up	Learn interpretable rules to predict recurrence after therapy	785 patients	Internal validation by train:test split of 80:20 (624:161), recurrences 42:11	Inductive Logic Programming (ILP) using DELMIA Process Rules Discovery, IF–THEN recurrence rules	Validation: sensitivity 71.4% (10/14), specificity 98.0% (144/147); 7/161 abstentions; AUC not reported
Jang et al., 2023	Multicenter observational cohort	Korea	pN1 PTC and ^131^I remnant ablation	Derive data-driven LN cutoffs to refine recurrence risk stratification	1232 patients: NED group (n = 1007): primary tumor size (cm, mean ± SD) = 1.8 ± 1.1, multifocality (n = 423), bilaterality (n = 302), microscopic extrathyroidal extension (n = 708), lymphatic invasion (n = 185), vascular invasion (n = 76), N1a (n = 645), N1b (n = 362), ipsilateral CCND (n = 463), bilateral CCND (n = 182), ipsilateral mRND (n = 242), bilateral mRND (n = 120); ^131^I activity (MBq): 1110 (n = 46), 2960 (n = 246), 3700 (n = 328), 5550 (n = 387); extranodal extension (n = 225), maximal diameter of metastatic LN foci (cm, mean ± SD) = 0.6 ± 0.6, number of retrieved LN (mean ± SD) = 17.5 ± 13.2, number of metastatic LN (mean ± SD): 4.1 ± 3.6, metastatic LN ratio (mean ± SD): 0.3 ± 0.2; Recurrence group (n = 225):, primary tumor size (cm, mean ± SD) = 2.5 ± 1.5, multifocality (n = 125), bilaterality (n = 88), microscopic extrathyroidal extension (n = 188), lymphatic invasion (n = 58), vascular invasion (n = 30), N1a (n = 131), N1b (n = 94), ipsilateral CCND (n = 80), bilateral CCND (n = 51), ipsilateral mRND (n = 57), bilateral mRND (n = 37); ^131^I activity (MBq): 1110 (n = 5), 2960 (n = 36), 3700 (n = 62), 5550 (n = 122); extranodal extension (n = 84), maximal diameter of metastatic LN foci (cm, mean ± SD) = 0.8 ± 0.8, number of retrieved LN (mean ± SD) = 17.7 ± 14.0, number of metastatic LN (mean ± SD): 6.4 ± 5.3, metastatic LN ratio (mean ± SD): 0.5 ± 0.3	Internal validation by train:test split of 70:30	Unsupervised ML with the K-means clustering algorithm; K-means to derive data-driven cutoff values for continuous lymph-node variables (e.g., maximal diameter of metastatic LN foci, number of metastatic LNs, and metastatic LN ratio) to form new LN-based risk categories for recurrence-free survival; authors paired this with conventional survival statistics (restricted cubic splines in a Cox model to show nonlinearity, and Kaplan–Meier/log-rank comparisons)	New cutoffs: 0.2/1.1 cm (max. LN focus), 4/13 (metastatic LN), 0.28/0.58 (LN ratio); high-risk categories independently predicted recurrence (HRs reported); no AUC
Shrestha et al., 2025	Retrospective database	Malaysia Bangladesh	DTC: PTC, micro-PTC, FTC, Hürthle cell carcinoma	Improve recurrence prediction via metaheuristic optimization of XGBoost	383 patients from dataset collected from the University of California, Irvine (UCI); recurrence cases (n = 120), PTC (n = 287), micro-PTC (n = 48), FTC (n = 28), Hürthle cell carcinoma (n = 20); stage I (n = 333), stage II (n = 32), stage III (n = 4), stage IVA (n = 3), stage IVB (n = 11); T1a (n = 49) and T1b (n = 43), T2 (n = 149), T3 (n = 110),including T3a (n = 94), T3b (n = 16), T4 (n = 26), T0 (n = 0); nodal status showed N0 (n = 264), N1 (n = 119), M0 (n = 365), with M1 (n = 18)	External validation on an unseen dataset, no detailed characteristics	XGBoost with hyperparameter tuning using WOA + modified WOA (chaotic init + inertia); one-hot encoding; optional feature selection	Model has shown an accuracy of 99% when optimized with WOA and 97% accuracy when optimized with the modified WOA
Deif et al., 2025	Retrospective single-center	United Arab Emirates/Egypt	DTC recurrence after RAIT	Optimize recurrence prediction “after RAIT” using a new metaheuristic + XGBoost	383 DTC (PTC + FTC) patients; recurred (n = 108`0, no recurrence (n = 275)	Internal cross-validation	TIEO-XGBoost: Tribal Intelligent Evolution Optimization for XGBoost hyperparameters; compared vs GA/PSO/SA/LP-GA; fivefold CV	AUC 0.931, accuracy 0.889, precision 0.851, recall 0.867, F1 0.859; Wilcoxon *p* < 0.05
Triayudi et al., 2025	Retrospective database	Indonesia	Thyroid cancer of mixed histologies	Interpretable recurrence prediction + risk stratification for follow-up planning	Three complementary data sources were integrated into the final cohort (n = 22002): Dharmais (n = 165), TCGA-THCA (n = 472), SEER (n = 21365); no detailed characteristics	External and multicenter validation, no detailed characteristics	Interpretable hybrid: AMSFCoC-DMU fuzzy co-clustering + HKD-CSC (teacher models distilled to calibrated logistic-regression “student”; cost-sensitive calibration)	Proposed model: AUC 0.963, accuracy 0.948, recall 0.938, Brier 0.061; external validation AUC 0.951 (TCGA/SEER)

Abbreviations: ^*131*^*I-MIBG* ^131^I metaiodobenzylguanidine, ^*131*^*I-WBS* ^131^I whole-body scan, *A/P* anterior/posterior (counts/views), *AMSFCoC-DMU* Adaptive Multi-Source Fuzzy Co-Clustering with Dynamic Membership Updating, *ANN* artificial neural network, *AST* aspartate aminotransferase, *ATA* American Thyroid Association, *AUC* area under the curve, *AUROC* area under the receiver operating characteristic curve, *BEf* bi-exponential function, *CCND* central compartment neck dissection, *CI* confidence interval, *CNN* convolutional neural network, *CV* cross-validation, *DELMIA* DELMIA software, *DSC* Dice similarity coefficient, *DT* decision tree, *DTC* differentiated thyroid cancer, *ER* excellent response, *F1* F1 score, *FPN* feature pyramid network, *GA* genetic algorithm, *GMDH* Group Method of Data Handling, *HD95* 95th-percentile Hausdorff distance, *HKD-CSC* Hybrid Knowledge Distillation with Cost-Sensitive Calibration, *HPO* hyperparameter optimization, *HR* hazard ratio, *ILP* inductive logic programming, *KM* Kaplan–Meier, *kNN/KNN* k-nearest neighbors, *L-T4* levothyroxine, *LASSO* least absolute shrinkage and selection operator, *LN* lymph node, *LP-GA* linear programming plus genetic algorithm, *LR* logistic regression, *MAE* mean absolute error, *MC* Monte Carlo, *MCNP* Monte Carlo N-Particle, *mDTC* metastatic differentiated thyroid cancer, *MEf* mono-exponential function, *MIRD* Medical Internal Radiation Dose, *ML1/ML2* model label 1 and model label 2, *MLP* multilayer perceptron, *MPA* maximum permissible activity, *mRND* modified radical neck dissection, *MSE* mean squared error, *MSRA-UNet* + + UNet + + variant, *N1* nodal stage N1, *NET* neuroendocrine tumor, *NM* nuclear medicine, *NN* neural network, *NED* no evidence of disease, *non-ER* non-excellent response, *OSS* one-sided selection, *PLS-DA* partial least squares discriminant analysis, *PSO* particle swarm optimization, *PTC* papillary thyroid carcinoma, *R-CNN* region-based convolutional neural network, *RF* random forest, *RFE* recursive feature elimination, *RMSE* root mean squared error, *ROC* receiver operating characteristic, *ROI* region of interest, *RR* risk ratio/relative risk, *Rx-WBS* post-therapy whole-body scan, *R*^*2*^ coefficient of determination, *SA* simulated annealing, *SE-Net* squeeze-and-excitation network, *SEER* Surveillance, Epidemiology, and End Results, *SHAP* SHapley Additive exPlanations, *SMOTE* Synthetic Minority Over-sampling Technique, *SPSS* Statistical Package for the Social Sciences, *SVM* support vector machine, *SVR* support vector regression, *TAC* time-activity curve, *TCGA* The Cancer Genome Atlas, *TCGA-THC* TCGA thyroid carcinoma cohort, *TIEO* Tribal Intelligent Evolution Optimization, *TIEO-XGBoost* XGBoost with TIEO hyperparameter optimization, *TMA* thyroid microsomal antibody, *UCI* University of California, Irvine, *UNet* + + U-Net + + architecture, *VGG-16* Visual Geometry Group 16-layer CNN, *VIP* variable importance in projection, *WB* whole body, *WBS* + whole-body scan positive, *WDTC* well-differentiated thyroid cancer, *WOA* whale optimization algorithm, *XGBoost* eXtreme Gradient Boosting

### Studies Characteristics

Across the 24 included studies, the most common design was retrospective single-center, sometimes based on synthetic data. Notably, 10 of the included studies were based on Chinese patient cohorts, 2 from Korea, 2 from Iran and 2 from the USA. Patient populations were typically female-predominant and middle-aged (most cohorts reporting mean ages in the 40 s–50 s), and the most frequent indication for RAIT was differentiated thyroid cancer, especially papillary thyroid carcinoma. Fewer studies addressed benign thyroid disease (e.g., Graves’/hyperthyroidism) or post-treatment management (e.g., hypothyroidism/levothyroxine dosing), as well as radiation-safety and dosimetry contexts. The most common AI approaches were supervised “tabular” machine learning models, most frequently using tree-based ensembles/boosting (random forest, gradient boosting/XGBoost/Extra Trees) plus SVM and logistic regression, while a smaller subset used DL on imaging (4 out of 24, mainly CNN/U-Net–type architectures for CT or whole-body scintigraphy tasks). A few studies emphasized interpretability/structure discovery via rule learning, clustering, or hybrid interpretable frameworks (3 out of 24 studies). Overall, studies’ aims clustered around decision support for predicting iodine avidity or non-avidity or occult metastases before therapy, forecasting treatment response/ablation success, recurrence or therapy failure, and enabling more personalized and efficient care through dosimetry or administered-activity planning, discharge and radiation-safety prediction, automated image classification, segmentation, and individualized levothyroxine dosing.

### Quality Assessment

**Fig. 3:**
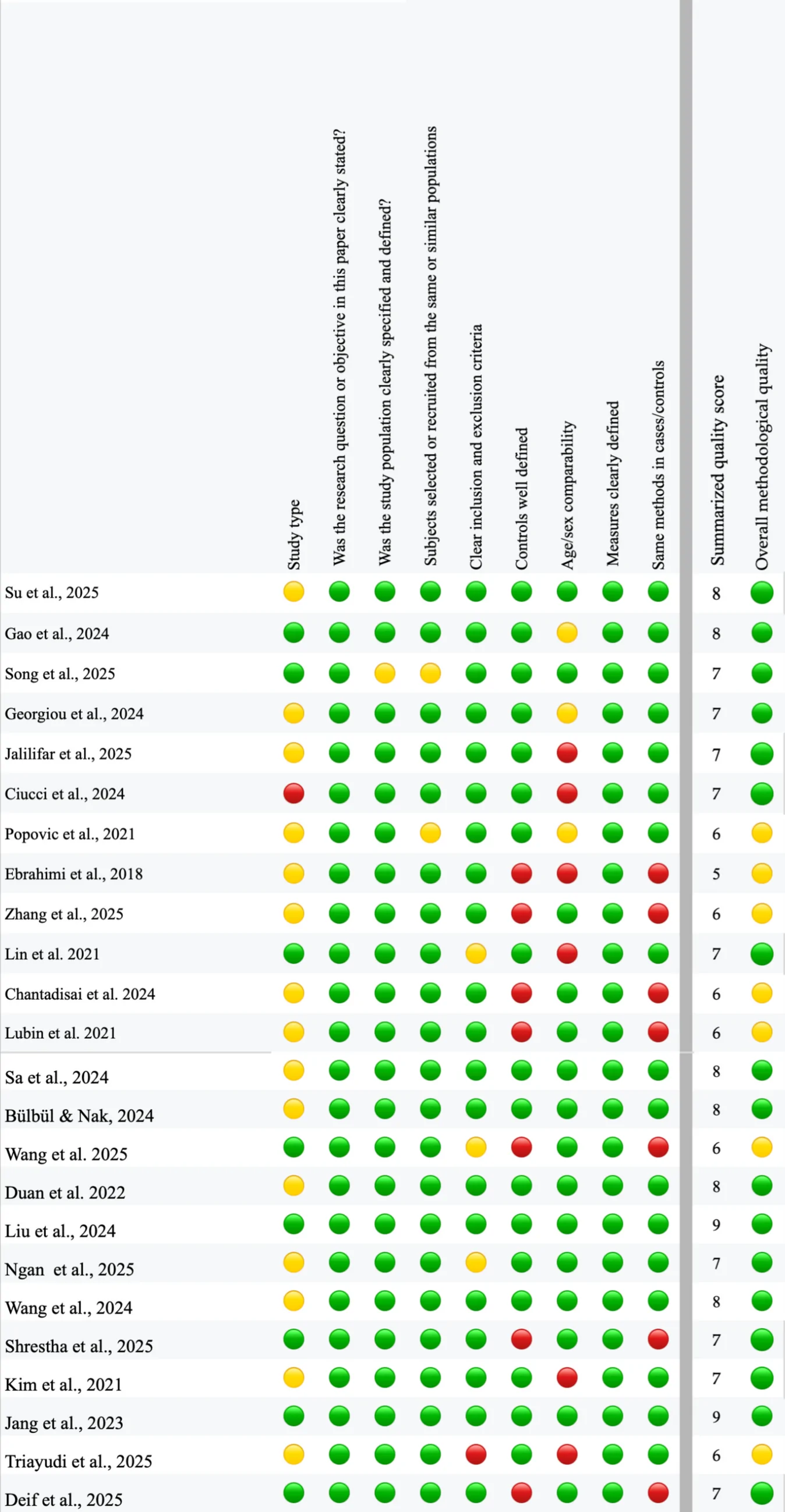
Quality assessment of the included studies

The methodological quality of the included studies was assessed using the Study Quality Assessment Tool developed by the National Heart, Lung, and Blood Institute (NHLBI), National Institutes of Health (NIH). Each criterion was rated as “Yes” (criterion fulfilled; green), “No” (criterion not fulfilled; red), or “Cannot Determine/Not Reported” (insufficient information; yellow). For quantitative assessment, fulfilled criteria (“Yes”) were assigned 1 point, whereas criteria rated as “No” or “Cannot Determine/Not Reported” were assigned 0 points. The overall score was calculated as the percentage of fulfilled applicable criteria. Studies were categorized as having high, moderate, or low methodological quality based on the proportion of fulfilled NIH/NHLBI criteria: ≥ 75%, 50–74%, and < 50%, respectively. The assessment was performed independently by two reviewers, and any disagreements were resolved through discussion and, when necessary, consultation with a third reviewer. The summarized quality assessment results are presented in Fig. 3.

### Therapy Planning

#### Selecting Patients

Selecting appropriate candidates for RAIT in differentiated thyroid cancer (DTC) is challenging because the clinical decision hinges on two distinct, but complementary, pre-therapy questions: whether a patient harbors clinically relevant metastatic disease that may be occult on conventional imaging yet detectable on ^131^I whole-body scanning, and whether identified metastases are likely to be ^131^I-avid (and therefore treatable) versus non-avid (^131^I-refractory), in which case additional ^131^I exposure may offer limited benefit [87, 88]. Su et al. (2025) study represents the first trend by creating an explainable, clinic-ready tabular ML by assembling a retrospective cohort of ^131^I-treated DTC patients and defining occult lung metastasis as CT-negative but ^131^I-WBS-positive disease [63]. They screened candidate predictors and retained 11 routine variables (demographics, staging/tumor features, LN metastasis count, TSH/Tg/TgAb, administered activity), trained six classifiers (SVM/DT/RF/LR/XGBoost/KNN) with a 7:3 train–test split, and used SHAP to interpret the best-performing LR model. Gao et al. (2024) [64] and Song et al. (2025) [65] reflect the second trend: CT-based deep learning as a surrogate for uptake phenotype, and the field’s move from single-step classifiers to workflow models. Gao used pretreatment non-contrast CT with avidity labels derived from post-RAIT SPECT, implementing a two-stage segmentation: classification cascade (SE V-Net then SE-Net). Song et al. built a multicenter pipeline that first detects nodules on pretreatment CT (Faster R-CNN/FPN) and then classifies uptake phenotype (ResNeSt50), trained and evaluated with cross-validation and independent internal/external testing using Rx-WBS as the reference label.

Beyond confirming the central role of Tg as a surrogate of disease burden, these AI studies highlight additional determinants relevant to selection, including demographics and basic clinicopathologic features. In Su et al. occult lung metastasis risk was independently associated with older age and male sex, alongside higher stage/invasion and thyroid biomarkers. In the CT-based uptake studies, age also differed by phenotype: Gao et al. reported older patients in the non-avid group (with no significant sex difference), and Song et al. similarly observed a slightly older non-avid group. Importantly, both imaging pipelines emphasize small nodule size as a key practical limiter for CT-driven triage, underscoring that the hardest lesions to detect are also the hardest to classify reliably. Together, they support a selection paradigm that aims to minimize futile exposure, streamline escalation to alternative strategies when appropriate, and move toward more personalized, uptake-informed treatment planning.

#### Protocols, Activity Selection, Dosimetry, and Biokinetics

Individualized ^131^I dosimetry can help maximize tumor dose while staying within safety limits (e.g., avoiding bone marrow toxicity and radiation pneumonitis), but “full” protocols are often time-consuming (multi-day imaging + repeated blood draws), which is a major barrier to routine use. Recent AI research in RAIT is converging on a common objective: enabling individualized dosimetry and activity selection despite practical constraints (limited sampling, limited imaging resources, and inter-physician variability). In Georgiou et al. (2024) [66], a feedforward deep-learning neural network (regression; two hidden layers) was trained to predict maximum permissible activity (MPA) from early 4/24/48-h measurements, achieving no significant difference versus the full MIRD protocol in an internal test set (*p* = 0.351), supporting protocol compression by omitting late time points. In Jalilifar et al. (2025) [67], multiple ML approaches were compared (including linear regression, SVR, decision tree), with a multilayer perceptron (MLP) performing best for predicting organ absorbed doses from planar imaging plus a single 45° oblique view, validated against SPECT/CT GATE Monte Carlo ground truth and further assessed in external validation; most organs showed < 15% differences, while spleen and lung regions retained larger negative biases, consistent with expected challenges in smaller/motion-affected structures. In Ciucci et al. (2024) [68], a two-stage ensemble was used to stabilize time-integrated activity (τ/AUC) under sparse/noisy sampling: ML1 (soft-voting logistic regression classifier) selected mono- vs bi-exponential kinetics, and ML2 (AdaBoost with gradient-boosting regressors) estimated parameters/τ. This approach improved robustness relative to conventional fitting with AICc/F-test selection (∼98% classification accuracy; τ errors were constrained to ± 25%), with the unexpected observation that ML frequently favored bi-exponential behavior in patient TACs while preserving high agreement in τ. Finally, Popovic et al. (2021) [69] applied supervised classification to postoperative planning, where an artificial neural network (ANN) best reproduced expert activity-category decisions (95.71%, κ = 0.96) and improved junior-physician concordance after ANN-assisted learning, highlighting utility as both decision support and a training/standardization tool. Overall, these studies suggest AI can support clinically actionable RAIT management by shortening dosimetry workflows, approximating high-fidelity dosimetry from accessible imaging, improving τ estimation with limited time points, and standardizing activity selection, although external validation remains variable and performance limitations cluster in anatomically or kinetically challenging cases.

#### Safety, Radiation Exposure and Outpatient Management

Radiation protection considerations after therapy extend beyond the treated individual to household contacts and also shape service delivery, because inpatient isolation capacity and discharge timing depend on residual activity and dose-rate constraints. Within this context, two included studies evaluated data-driven approaches to support release decisions and outpatient logistics.

Ebrahimi et al. (2018) [70] pursued individualized third-party dosimetry, training a feed-forward neural network on 52 thyroid cancer families (99 patient–relative pairs) using direct relative dose measurements (dosimeter worn 7–21 days) plus patient dose-rate kinetics (2–48 h) and standard release metrics. Despite generally low observed exposures (mean 0.45 mSv; 7 relatives > 1 mSv, none > 5 mSv), the network delivered an expected improvement over conventional patient-specific calculations and, more notably, an unexpectedly large gain in identifying higher-risk relatives (> 1 mSv: AUC 0.957 vs 0.633), highlighting a practical role for risk-stratified counseling and tailoring restrictions, especially in households with young children. Zhang et al. (2025) [71] addressed a complementary operational endpoint: 48-h discharge eligibility under a national residual-activity threshold (< 400 MBq). They fitted an exponential decay model to serial residual activity measurements obtained every 24-h at a distance of 1 m. This approach enabled estimation of the effective half-life. Subsequently, they trained an SVM model using readily available clinical and retention features. They reported strong discrimination (AUC 0.92; accuracy 88.04%) consistent with the mechanistic expectation that shorter effective half-life predicts earlier discharge, and identified sex and 24-h residual activity as influential predictors, supporting earlier planning of isolation duration while remaining aligned with safety limits.

### Therapy Assessment

#### Imaging Analytics

Lin et al. (2021) [72] exemplify the earlier workflow-oriented paradigm by training an attention-augmented CNN (Dscint) for whole-body scintigraphy classification (normal, bone metastasis, arthritis, thyroid carcinoma) using physician report–derived labels and an internal 7:3 patient-separated split (10 repeated runs). Notably, performance proved highly augmentation-dependent, deeper architectures (e.g., Inception-v4) did not improve discrimination, and class-averaged metrics masked clinically relevant failure modes (lowest F1 in arthritis), collectively highlighting data scarcity and label/phenotype heterogeneity as key constraints for robust deployment. In contrast, Chantadisai et al. (2024) [73] represent the emerging decision-support paradigm by coupling automated ROI-based radiomics with regularized logistic regression to predict successful ablation at 6–12 months after standardized low-risk PTC treatment, using cross-validated model selection and a clinically anchored composite outcome. Expectedly, radiomics alone did not outperform Tg, but the combined model improved discrimination primarily via higher specificity, while an important practical limitation, segmentation failure at near-background uptake, clustered in very low Tg, favorable-outcome cases, implying that “non-informative” images may themselves signal low risk.

Both studies on imaging differ in endpoint (diagnostic categorization vs ablation success), modeling paradigm (end-to-end DL vs radiomics + clinical integration), and dominant technical constraints (data augmentation dependence and class-specific error patterns vs segmentation/feature-extraction feasibility and incremental gains from combining imaging with Tg). However, together they support the feasibility of AI to either standardize scan interpretation or enhance outcome stratification from ^131^I imaging, while also underscoring the need for stronger generalizability evidence beyond internal validation.

#### Early and Intermediate Treatment Outcomes in DTC/PTC

Over the past few years, AI research in the RAIT pathway has evolved from describing isolated prognostic factors to building multivariable predictive decision-support models from routine clinical and biochemical data. In a small single-center cohort, Lubin et al. (2021) [74] used a random forest model based on EMR features to predict first RAIT failure. The identified predictors were largely expected and included higher pre-RAIT Tg/TgAb levels and adverse pathology findings. The model also showed an atypical association between higher administered activity and therapy failure. This association was most consistent with confounding by indication. Importantly, the ML approach identified predictors that were not retained in classical logistic regression models, illustrating that nonlinear models can recover signals missed by traditional approaches. Sa et al. (2024) [75] extended this trend with larger retrospective datasets and an explicitly interpretable pipeline (LASSO feature selection, multi-algorithm benchmarking, SHAP explanations) to predict effective response to RAIT and biochemical remission under TSH suppression, achieving strong internal discrimination and reinforcing that Tg metrics dominate prediction while adding actionable contributions from uptake or scan surrogates (eg, remnant on Rx-WBS) and TSH suppression adequacy. Bülbül & Nak (2024) [76] similarly applied multiple classifiers with imbalance handling and cross-validation in papillary thyroid cancer, finding that while few single variables differentiated ER vs non-ER (notably TgAb), boosting models still achieved good AUC/accuracy, supporting the recurring pattern that ML can integrate many weak predictors into clinically useful stratification.

#### Post-Therapy Endocrine Management

Recent AI work around ^131^I is expanding beyond imaging or dosimetry into post-therapy endocrine management, where the clinically decisive problem is how quickly and safely patients reach their individualized TSH targets on levothyroxine or restore euthyroidism after post-therapy hypothyroidism. Liu et al. (2024) [77] exemplify a trend toward lean, clinic-ready models with prospective utility. Using random-forest–guided selection from 16 candidates, they retained six simple predictors (BSA, weight, hemoglobin, height, BMI, age) and found SVR to perform best. In a two-center prospective implementation, the model-predicted dose was initiated on day 2 after RAIT. This approach markedly increased early attainment of ATA risk-adapted TSH targets and shortened the time-to-target compared with historical empirical dosing. These findings demonstrate a clinically meaningful benefit of decision support, even though the per-dose step accuracy was only moderate. In contrast, Ngan et al. (2025) [78] reflect the parallel trend of high-dimensional EMR modeling in broader hypothyroidism populations (including post-RAIT cases), integrating comorbidities, drug–drug interactions, food/timing effects, etiology, and labs. An Extra Trees regressor achieved strong internal dose-prediction performance, with BMI and comorbidity burden emerging as dominant drivers, which is an expected finding biologically, but notable in quantifying that these factors may outweigh simple weight-based dosing.

Collectively, a key next research step would be development and external validation of models that jointly predict dose plus individualized monitoring cadence (i.e., when to remeasure thyroid function and revisit) to operationalize truly personalized post-RAIT endocrine follow-up.

### Benign Hyperthyroidism Applications

Despite the predominance of AI research in differentiated thyroid cancers, optimizing RAIT management in non-malignant thyroid disorders remains clinically important, as hyperthyroidism is highly prevalent and can be successfully treated with ^131^I, motivating AI approaches that improve planning and follow-up [41, 42].

Duan et al. (2022) [79] retrospectively analyzed Graves’ disease patients receiving first RAIT and applied ML to predict hypothyroidism at 6 months using 138 baseline clinical and laboratory variables. Feature selection combined random-forest importance, partial least squares discriminant analysis VIP scores, and Mann–Whitney testing with stepwise refinement to derive a 7-variable model (age, thyroid mass, 24-h ^131^I uptake, AST, TRAb, TMA, neutrophil count) presented as a nomogram. At 6 months, 69.4% developed hypothyroidism, and model discrimination was moderate with an area under the receiver operating characteristic curve of 0.72 in training and 0.74 in validation, supporting individualized post-treatment surveillance and earlier planning for thyroid hormone replacement. In a complementary application focused on enhancing accuracy and reliability in absorbed dose calculations, Wang et al. (2025) [80] developed an AI-based tool to automate thyroid gland segmentation on CT scans and use the resulting geometry to calculate patient-specific radiation doses in hyperthyroid patients treated with radioiodine. Their model (MSRA-UNet + +) outperformed the standard UNet + + approach in segmentation accuracy (Dice score 85.61% vs. 81.15%) and additionally generated uncertainty maps to flag ambiguous regions for clinician review. When AI-derived segmentations were used for dosimetry, the traditional Marinelli–Quimby formula overestimated absorbed dose by an average of 7.74% (up to 10.73%) compared to the more precise MIRD/Monte Carlo method, suggesting that geometry-aware dose calculation could meaningfully improve activity planning in benign thyroid radioiodine therapy.

### Long-Term Follow-up: Recurrence Prediction and Risk Stratification

Investigations applying AI to predict outcomes after RAIT in DTC have largely focused on post-treatment recurrence risk stratification and on employing diverse methodological paradigms including supervised predictive modeling, symbolic rule induction, unsupervised cutoff discovery, and metaheuristic optimization. Supervised tabular ML in real-world institutional cohorts shows modest but credible gains: Wang et al. (2024) [81] gathered 29 potential input variables and trained LR/SVM/XGBoost/RF/NN with imbalance handling, LASSO feature selection, and CV/Bayesian tuning, achieving only moderate discrimination, yet outperforming ATA stratification and reinforcing expected dominant signals from postoperative Tg and nodal burden, while adding a potentially novel contribution from metabolic comorbidities. In parallel, Kim et al. (2023) [82] used inductive logic programming to generate interpretable IF–THEN rules driven largely by serial Tg, yielding high specificity but lower sensitivity and abstentions, consistent with a role in dynamic re-stratification rather than early prediction. Jang et al. (2023) [83] applied K-means clustering with repeated resampling to derive data-driven lymph-node cutoffs that separated recurrence-free survival (HR-based risk groups), illustrating an alternative “AI-as-cutpoint-discovery” paradigm without AUC-based classification. In contrast, studies using the public UCI datasets, such as the studies by Shrestha et al. (2025) on WOA–XGBoost [84] and Deif et al. (2025) on TIEO–XGBoost [85], report very high performance under cross-validation, which, while methodologically focused on optimizer novelty, appears less clinically persuasive given the absence of external validation and potential optimism. Triayudi et al. (2025) [86] most directly addressed generalizability by combining fuzzy co-clustering with cost-sensitive distillation to a calibrated student model and validating across TCGA/SEER, supporting feasibility of portable recurrence-risk models. Clinically, these approaches collectively suggest a pathway toward more individualized post-RAIT surveillance intensity; however, prospective multicenter evaluation with therapy-specific inputs remains the key gap.

## Discussion

### Categorization of AI Methods by Clinical Utility and Modeling Approach

Across the 24 included studies, artificial intelligence methodologies were applied across the full RAIT care continuum, encompassing pre-therapy assessment, treatment planning, radiation safety and logistical management, as well as post-therapy evaluation and long-term follow-up. This comprehensive distribution of applications suggests that AI may contribute to a clinical paradigm shift in RAIT by moving from isolated, task-specific analytical tools toward an integrated, end-to-end decision-support framework embedded within routine workflows across the entire patient pathway.

**Fig. 4:**
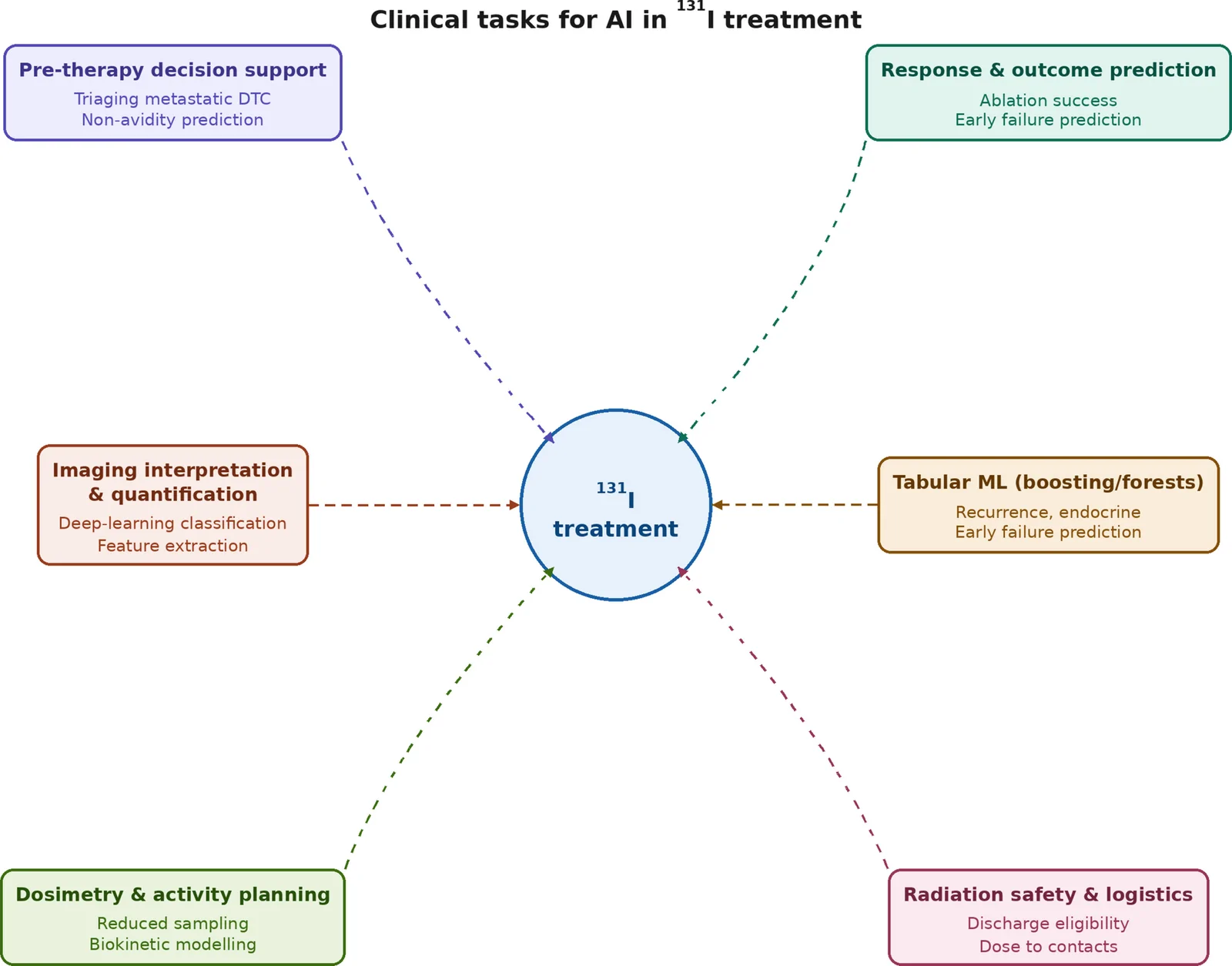
Clinical tasks for AI in ^131^I treatment

Furthermore, AI applications in RAIT can be systematically grouped by clinical tasks along the care pathway into: (i) pre-therapy decision support (e.g., triaging metastatic DTC such as predicting iodine avidity/non-avidity from pretreatment imaging or clinicopathologic variables), (ii) imaging interpretation and quantification (deep-learning classification/standardization of whole-body scintigraphy and post-therapy scan feature extraction), (iii) response and outcome prediction (ablation success, excellent/effective response, or early therapy failure using radiomics + clinical inputs or tabular ML), (iv) dosimetry and activity planning (NN/ML surrogates to reduce sampling burden, approximate Monte Carlo dosimetry from pragmatic planar protocols, and model biokinetics under sparse measurements), (v) radiation safety and logistics (predicting discharge eligibility and exposure to relatives/contacts), and (vi) downstream management and surveillance (levothyroxine dosing support and recurrence-risk stratification). See Fig. 4.

### Data Sources and Validation Strategies

In the included studies, AI models for RAIT drew on heterogeneous data streams that can be summarized into four recurrent input domains. First, nuclear medicine imaging (whole-body scintigraphy, post-therapy scans, and in some workflows planar/SPECT-derived measurements) was used both as a direct prediction substrate (e.g., DL-based scan classification) and as a source of quantitative imaging features (radiomics or uptake-derived metrics). Second, anatomic imaging, predominantly CT, served as the key modality for models targeting metastatic characterization and iodine avidity or non-avidity phenotyping. Third, most predictive models incorporated a broad set of clinical, demographic, and laboratory variables beyond thyroid-specific biomarkers, commonly including age, sex, anthropometrics (BMI, weight, body surface area), cardiovascular, metabolic markers (e.g., lipid profile such as LDL), renal and hepatic function surrogates, medication history, and comorbidity burden, alongside disease-specific parameters (TNM/ATA features, nodal burden, administered activity, TSH suppression, and Tg/TgAb dynamics). Fourth, dosimetry and biokinetic datasets (serial activity or dose-rate measurements, blood sampling, effective half-life, and time–activity curve parameters) were used in studies aiming to scale individualized activity planning or approximate Monte Carlo dosimetry under pragmatic sampling constraints. Overall, this heterogeneity of input data highlights that AI-driven RAIT models are inherently multimodal, integrating both functional and anatomical imaging.

### Methodological and Clinical Trends

Several consistent methodological and clinical patterns emerge from the included studies. Clinically, the literature is strongly skewed toward DTC, particularly papillary thyroid cancer, with comparatively fewer models addressing benign thyroid diseases. The most frequently targeted endpoints were recurrence or structural disease progression, followed by treatment response–related outcomes (e.g., ablation success, excellent/effective response, or early therapy failure), reflecting the central role of RAIT in long-term oncologic surveillance rather than acute decision-making. In contrast, fewer studies focused on operational or safety-related endpoints, such as discharge eligibility or radiation exposure of contacts, despite their high practical relevance. Methodologically, there is a clear division by data type: deep learning dominates image-centric tasks (e.g., scan classification, lesion detection, uptake phenotyping), whereas tabular machine learning approaches (often tree-based ensembles or gradient boosting) prevail in recurrence prediction, endocrine dose estimation, and risk stratification. Importantly, a growing subset of studies emphasizes interpretability and clinical transparency, using rule-based systems, SHAP explanations, or distillation-based frameworks, signaling a shift away from purely black-box models toward decision-support tools. This provides evidence of a gradual transition from retrospective model development toward clinically actionable decision-support systems that can be integrated into routine RAIT workflows to support treatment personalization, improve consistency of dosing and follow-up strategies, and ultimately enhance patient safety and therapeutic outcomes. In the longer term, such tools may also help reduce the routine workload and administrative burden on clinical staff; however, they are intended to complement rather than replace specialist expertise in nuclear medicine.

### Barriers to Implementation and Future Directions

Across the included studies, the main barriers to clinical implementation of AI in RAIT cluster into issues of generalizability, interpretability, workflow integration, and governance. Many models are trained on retrospective, single-center datasets with scanner-, protocol-, and population-specific characteristics, which increases the risk of dataset shift and performance degradation when transferred to new institutions or clinical settings. Moreover, the predominance of Chinese cohorts among the included studies may influence the representativeness of the findings, as thyroid disease epidemiology and management differ across geographic regions. China has historically experienced both iodine deficiency and iodine excess despite long-term universal salt iodization programs, resulting in regional variability in iodine exposure and thyroid disease prevalence [87]. Differences in dietary iodine intake, environmental exposure, TSH reference ranges, and treatment practices may affect the clinical characteristics of thyroid disorders, potentially influencing AI model development and predictive performance, therefore, more studies across diverse geographic populations are needed, and ideally, future AI models should be developed using heterogeneous multinational patient cohorts to improve external validity and reduce population-specific bias [30].

Furthermore, endpoint and label heterogeneity (e.g., varying definitions of ablation success, response categories, recurrence, or iodine avidity) limit reproducibility and make benchmarking difficult, while label quality can be constrained by imperfect reference standards and follow-up variability [88]. Moreover, 6–24 months evaluation windows used in many studies can introduce a time-window (follow-up duration) bias, because late events such as DTC recurrence or RAIT-induced hypothyroidism may be under-ascertained when outcomes are assessed within a short period of time [89, 90].

In addition, several approaches face practical workflow constraints, such as failure of segmentation or feature extraction when uptake is low, reduced sensitivity for small lesions, or reliance on dosimetry and biokinetic inputs that are not routinely collected, meaning that high-performing models may not be feasible at scale. Furthermore, implementation requires more than discrimination metrics: many studies provide limited reporting on calibration, uncertainty, and decision thresholds, and only rarely evaluate clinical impact, safety trade-offs, or how clinicians should act on model outputs.

Importantly, despite the fact that RAIT is widely used in benign thyroid disorders, most current AI research is concentrated on thyroid cancer, leaving a substantial opportunity gap. In benign disease, the clinical objective varies, but achieving and maintaining euthyroidism while minimizing post-therapy hypothyroidism creates a strong rationale for AI tools that can integrate high-dimensional clinical information to predict the patient-specific activity most likely to meet that goal. Because dosing in benign thyroid disease is frequently individualized and practice patterns vary, a robust, well-calibrated model that converges multimodal predictors into a transparent activity recommendation could meaningfully reduce trial-and-error dosing and support clinicians with consistent decision-making [91].

Although the educational role of AI in nuclear medicine was not directly evaluated in the reviewed studies, several papers compared the performance of AI predictors with that of human specialists. This suggests that future research may also explore AI-based educational and decision-support tools for training young physicians and improving clinical consistency [92]. Future directions could also address emerging applications of large language models (LLMs) in nuclear medicine, although current studies are still preliminary, LLMs could support research workflows and patient-facing communication tools (e.g., simplifying and translating RAIT instructions and improving risk–benefit communication), provided that rigorous validation and safety governance are established [93].

A further limitation is the use of the NIH/NHLBI Study Quality Assessment Tool rather than AI-specific frameworks such as PROBAST-AI or TRIPOD + AI [94, 95]. This decision was driven by the substantial methodological heterogeneity of the included studies, many of which were retrospective, exploratory, or focused on technical AI applications (e.g., segmentation, CT-free SPECT, dosimetry, and workflow optimization) rather than formal clinical prediction models. In addition, several studies lacked the detailed methodological reporting required for comprehensive AI-specific bias assessment. Therefore, a more general quality-assessment framework was considered more feasible for consistent cross-study evaluation in this broad systematic review [96]. With further maturation of the field, increasing availability of standardized reporting, and greater methodological sophistication of AI-based studies, future systematic reviews may consider the application of AI-specific risk-of-bias assessment frameworks whenever feasible and appropriate, to enable a more detailed evaluation of model development, validation, and potential sources of bias.

Ethical and governance considerations remain central to the safe integration of artificial intelligence into RAIT. Key issues include bias and fairness, particularly the risk of differential model performance across patient subgroups categorized by sex, age, body habitus, or disease phenotype, which may translate into inequities in clinical decision support. Data privacy and compliance with regulatory standards for sensitive health information are also essential, especially given the increasing use of multimodal datasets [97]. In addition, model drift over time (driven by changes in patient populations, imaging protocols, or treatment practices) poses challenges for maintaining long-term reliability and requires continuous monitoring and recalibration [98]. Importantly, because AI systems may influence decisions related to radiation exposure, administered activity, or treatment escalation, their use must be accompanied by transparent, auditable oversight mechanisms that ensure traceability of recommendations and preserve clinician accountability in final therapeutic decisions [97].

Alongside these technical challenges, ethical considerations remain central: clinicians and health systems must determine how much influence AI outputs should have, how to preserve clinical responsibility and informed consent, and how to prevent automation bias, especially in high-stakes contexts involving radiation exposure. More broadly, it is reasonable to ask whether, and under what safeguards, further AI development should be pursued. The strongest justification is when research is explicitly designed to improve patient outcomes, equity, and safety through transparent evaluation, prospective validation, and clear governance structures rather than pursuing performance metrics alone.

## Conclusion

Overall, the reviewed studies suggest that AI should currently be positioned as a clinical decision-support tool rather than a replacement for nuclear medicine specialists. The greatest immediate clinical value of AI appears to lie in supporting repetitive, data-intensive, and multidimensional tasks, such as imaging interpretation, risk stratification, dosimetry estimation, treatment-response prediction, and long-term surveillance. In this context, AI may improve workflow efficiency, standardization, and personalization of RAIT, while still requiring physician oversight and final clinical interpretation.

At present, however, most available evidence remains retrospective and methodologically heterogeneous, with limited external validation and inconsistent endpoint definitions. Therefore, the field has not yet reached the stage of fully autonomous clinical implementation. Future progress will likely depend on the development of multicenter prospective studies, harmonized reporting standards, explainable AI frameworks, and robust validation across different populations and imaging protocols.

Future AI applications in nuclear medicine will probably become increasingly embedded into routine workflows, assisting not only in prediction and planning, but also in therapy optimization, safety management, and physician training. At the same time, broader implementation will require careful attention to interpretability, calibration, regulatory oversight, and clinical accountability to ensure safe and trustworthy use in real-world practice.

## Data Availability

No new datasets were generated or analyzed in this study. Data sharing is not applicable to this article.

## Declarations

### Preprint sharing

No preprint version of this manuscript has been posted or is under consideration elsewhere.
